# Supplementary material for: Identification of Meloidogyne panyuensis (Nematoda: Meloidogynidae) infecting Orah (Citrus reticulata Blanco) and its impact on rhizosphere microbial dynamics: Guangxi, China
Source: PeerJ. 2024 Nov 6;12:e18495. doi: 10.7717/peerj.18495 (PMC11549905; doi:10.7717/peerj.18495)
Supplement: Supplemental Information 1 [file peerj-12-18495-s001.docx]

**Table S1. The raw data for the diameter (Fig 1D) and the mass (Fig 1E) of fruits.**
