# Supplementary material for: Identification of Meloidogyne panyuensis (Nematoda: Meloidogynidae) infecting Orah (Citrus reticulata Blanco) and its impact on rhizosphere microbial dynamics: Guangxi, China
Source: PeerJ. 2024 Nov 6;12:e18495. doi: 10.7717/peerj.18495 (PMC11549905; doi:10.7717/peerj.18495)
Supplement: Supplemental Information 2 [file peerj-12-18495-s002.docx]

| Number | Samples | pH | Organic matter（g/kg） | AN  （mg/kg） | AP  （mg/kg） | AK  （mg/kg） | TN  （g/kg） | TP  （g/kg） | TK  （g/kg） |
| --- | --- | --- | --- | --- | --- | --- | --- | --- | --- |
| 1 | CRH1 | 4.29 | 27.34 | 117.76 | 67.98 | 180.09 | 1.37 | 1.38 | 2.34 |
| 2 | CRH2 | 4.69 | 23.92 | 152.03 | 52.20 | 294.75 | 1.54 | 1.27 | 2.30 |
| 3 | CRH3 | 6.08 | 18.04 | 102.06 | 13.49 | 171.43 | 1.29 | 1.25 | 2.37 |
| 4 | CRN1 | 4.84 | 38.36 | 142.96 | 418.40 | 449.24 | 1.85 | 2.27 | 3.18 |
| 5 | CRN2 | 5.24 | 36.03 | 137.69 | 167.81 | 399.79 | 1.94 | 1.80 | 2.82 |
| 6 | CRN3 | 5.11 | 36.61 | 144.73 | 244.48 | 442.17 | 1.97 | 1.88 | 3.14 |

**Table S2. The raw data of soil chemical properties of the healthy and *M. panyuensis*-infected Orah rhizosphere soil (Table 1).**
