# Supplementary figures and images for: Identification of Meloidogyne panyuensis (Nematoda: Meloidogynidae) infecting Orah (Citrus reticulata Blanco) and its impact on rhizosphere microbial dynamics: Guangxi, China
Source: PeerJ. 2024 Nov 6;12:e18495. doi: 10.7717/peerj.18495 (PMC11549905; doi:10.7717/peerj.18495)

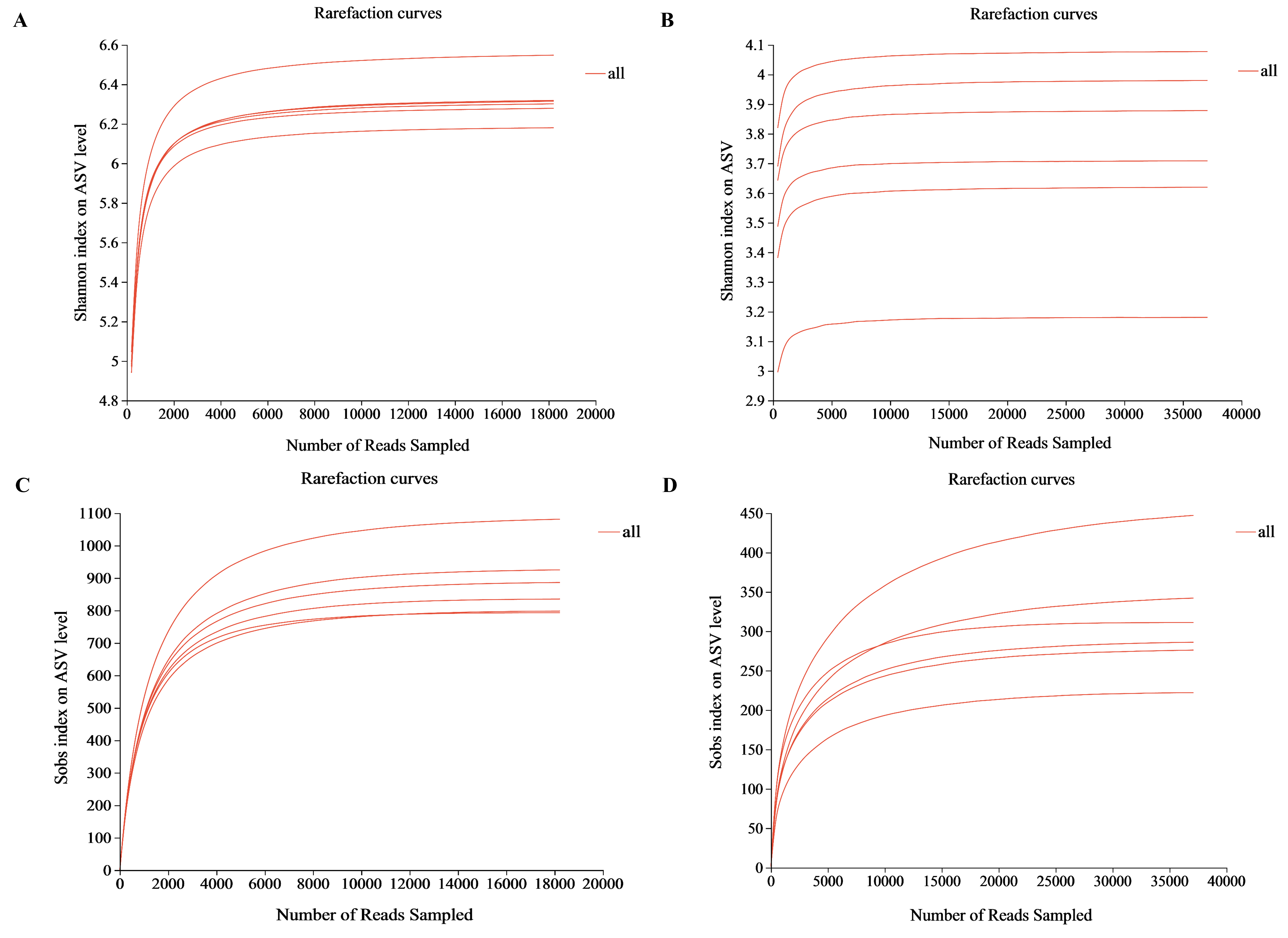

Supplement: Supplemental Information 3 — A (Shannon index) and C (Sob index) were the bacterial community; B (Shannon index) and D (Sob index) were the bacterial community. [file peerj-12-18495-s003.png]
